# Prophylactic senolytic treatment in aged mice reduces seizure severity and improves survival from *Status Epilepticus*

**DOI:** 10.1111/acel.14239

**Published:** 2024-06-20

**Authors:** Tahiyana Khan, Abbas I. Hussain, Timothy P. Casilli, Logan Frayser, Michelle Cho, Gabrielle Williams, David McFall, Patrick A. Forcelli

**Affiliations:** ^1^ Interdisciplinary Program in Neuroscience Georgetown University Washington District of Columbia USA; ^2^ Department of Pharmacology and Physiology Georgetown University Washington District of Columbia USA

**Keywords:** aging, Dasatinib, hippocampus, pilocarpine, quercetin, seizures, senolytic, *Status Epilepticus*

## Abstract

Increased vulnerability to seizures in aging has been well documented both clinically and in various models of aging in epilepsy. Seizures can exacerbate cognitive decline that is already prominent in aging. Senescent cells are thought to contribute to cognitive impairment in aging and clearing senescent cells with senolytic drugs improves cognitive function in animal models. It remains unclear whether senescent cells render the aged brain vulnerable to seizures. Here, we demonstrate that prophylactic senolytic treatment with Dasatinib and Quercetin (D&Q) reduced both seizure severity and mortality in aged C57BL/6J mice. We subjected the D&Q and VEH‐treated aged mice to spatial memory testing before and after an acute seizure insult, *Status Epilepticus [SE]*, which leads to epilepsy development. We found that senolytic therapy improved spatial memory before injury, however, spatial memory was not rescued after *SE*. Senescence‐related proteins p16 and senescence‐associated β‐galactosidase were reduced in D&Q‐treated aged mice. Our findings indicate that senescent cells increase seizure susceptibility in aging. Thus, prophylactically targeting senescent cells may prevent age‐related seizure vulnerability.

## Graphical abstract

Senescent cells accumulate in the brain with old age and contribute to cognitive dysfunction. Additionally, the risk for seizure vulnerability increases with old age. Our study hypothesized that accumulated senescent cells contribute to seizure vulnerability and cognitive decline in old age. We found that clearing senescent cells with senolytic cocktail, Dasatinib and Quercetin, prior to inducing seizures reduced seizure vulnerability and senescent cells in the hippocampi of aged mice. Created with Biorender.com (EH26RVMMFO).

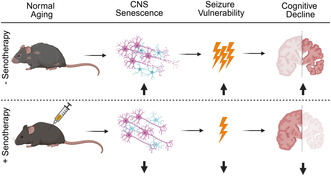

**Abbreviations**
BM: Barnes Maze
D&Q: Dasatinib and Quercetin
MWM: Morris Water Maze
PILO: Pilocarpine
SASP: Senescence‐associated secretory phenotype
SE: *Status Epilepticus*

## 1 INTRODUCTION

Individuals with advanced age represent the fastest‐growing group with epilepsy. Epilepsy is characterized by recurrent seizures as well as cognitive comorbidities (Stafstrom & Carmant, 2015). Epilepsy in older adults can arise secondary to disorders, including cerebrovascular disease (Liu et al., 2016) and neurodegenerative diseases, such as Alzheimer's disease (Sen et al., 2020). Although there is often a successful response to anti‐seizure medications in new‐onset epilepsy in older populations, side effects and drug–drug interactions may be exacerbated compared to younger populations (Anderson & Hakimian, 2018). Moreover, epilepsy can further exacerbate age‐related cognitive impairment in older adults with temporal lobe epilepsy (Reyes et al., 2021). The mechanisms resulting in enhanced sensitivity to seizures in aged populations are complex and remain incompletely understood.

A major hallmark of aging is cellular senescence, a process characterized by cell cycle arrest, resistance to apoptosis, and secretion of inflammatory factors (Gorgoulis et al., 2019). The upregulated production of pro‐inflammatory factors, often referred to as the senescence‐associated secretory phenotype (SASP) (Gorgoulis et al., 2019) includes release of factors such as IL‐β, TNF‐α, and matrix metalloproteases (Sun et al., 2022). Interestingly, many of these same factors are upregulated in epilepsy (Klein et al., 2018). Senescence triggers include oxidative stress, DNA damage, inflammation, and upregulation of cell cycle inhibitors, p53/p21^CIP1/WAF1^ and p16^INK4a^ (Baker & Petersen, 2018). Senescent cells have been associated with cognitive dysfunction and age‐related neurodegeneration (Bussian et al., 2018; Chinta et al., 2018; Ogrodnik et al., 2021; Xu et al., 2018; Zhang et al., 2019). Senolytic therapy (agents that eliminate senescent cells) reduces dysfunction in models of Alzheimer's and Parkinson's diseases and metabolic disorders (Bussian et al., 2018; Ogrodnik et al., 2021; Zhang et al., 2019), and has progressed to early phase clinical trials for non‐CNS indications (Nambiar et al., 2023).

Two of the most studied senolytic drugs, Dasatinib (D), a tyrosine kinase inhibitor that is an anticancer drug, and Quercetin (Q), a plant flavanol that inhibits mTOR and PI3K, are used in combination for senolysis. The D&Q combination significantly improves physiological functions in idiopathic pulmonary fibrosis (IPF) patients (Justice et al., 2019; Nambiar et al., 2023) and can effectively eliminate p16^INK4a^‐positive cells, reduce the activity of SA‐β‐gal, and attenuate the release of inflammatory factors in patients with diabetic nephropathy (Hickson et al., 2019). Quercetin alone has been shown to be neuroprotective in epilepsy models with little to no side effects (Diniz et al., 2015), however, they have not been explored in the context of aging and epilepsy. It is well recognized that senescent cells accumulate with aging and that aged populations have a high incidence of seizures and epilepsy (del Pozo et al., 2022). However, no study has assessed a link between senescence and epilepsy. Our study aimed to assess whether senescent cell elimination reduces vulnerability to seizures in aged mice.

## 2 METHODS

### 2.1 Experimental design and overview

We compared behavioral outcomes (Barnes Maze) and seizure susceptibility in aged (18‐month‐old) mice treated with a vehicle or a senolytic cocktail (D&Q) to that of young mice (5‐month‐old). Aged mice were treated with D&Q or vehicles from 14 to 18 months. Following seizure testing, animals received an additional 2 months of D&Q and were re‐tested for behavioral outcomes (Morris Water Maze [MWM]). At the conclusion of the experiments, animals were euthanized for histopathological assessment of senescence markers.

### 2.2 Animals

Twenty‐eight male and female young (5‐month‐old) and 56 aged (14‐month‐old) male and female C57BL/6 mice were obtained from Jackson Laboratory (Maine, USA). They were housed at 22°C with a 12 h light/dark cycle and allowed for free access to food and water. All experiments and procedures were carried out in accordance with the Guide for the Care and Use of Laboratory Animals (National Research Council (U.S.) et al., 2011), under a protocol approved by the Georetown Univeristy Animal Care and Use Committee (2019‐0037).

### 2.3 Treatment

Aged animals were randomized to receive pretreatment of senolytic cocktail, Dasatinib (i.p., 5 mg/kg, Selleckchem, S1021) Quercetin (i.p, 50 mg/kg, Sigma‐Aldrich, Q4951), or vehicle once a week from 14 months of age until the end of the experiment. Mice were pretreated with D&Q (*n* = 24) or vehicle (*n* = 32) for 16 weeks before seizure induction. Then, mice continued to receive treatment until the end of the experiment.

### 2.4 Seizure induction

We used the pilocarpine (PILO) model of epilepsy (Turski et al., 1984). Pilocarpine is a chemoconvulsant that is used to induce *Status Epilepticus* (SE), in which prolonged seizures cannot self‐terminate. Animals were pretreated with scopolamine methyl bromide (Sigma‐Aldrich, S8502) and terbutaline hemi sulfate (Sigma Aldrich, T2528) (both i.p., 2 mg/kg) to block peripheral effects of PILO and dilate the respiratory tract. Thirty minutes after, 260 mg/kg PILO hydrochloride (Cayman Chemicals, c23131841) was administered intraperitoneally. Acute seizures were behaviorally monitored using the following seizure rating scale (stage 1, facial clonus; stage 2, unilateral forelimb clonus; stage 3, bilateral forelimb clonus; stage 4, rearing with forelimb clonus; stage 5, rearing and falling with forelimb clonus; stage 6, jumping and running). SE was defined by 5 min of stage 3 continuous tonic‐clonic convulsive seizures. Mice were monitored for 2 h until seizure activity was reduced with diazepam (i.p., 5 mg/kg; Dash Pharmaceuticals). After the termination of SE, mice were administered 5% dextrose solution to facilitate their recovery and improve survival.

### 2.5 Behavior

Behavioral tests were conducted before (i.e., after 4 months of senolytic treatment in 18‐month‐old mice) and after epilepsy development (i.e., 6 months of senolytic treatment in 20‐month‐old mice). Testing occurred both prior to SE to assess the effects of senolytic therapy on baseline memory function in aged mice and 2 months after SE (a time at which epilepsy is expected to have developed) to assess senolytic therapy on memory function after SE. Cognitive deficits are prominent with SE and epileptogenesis, and spatial memory deficits have been reported both in aged mice (Barnes, 1979; Barreto et al., 2010; Hendrickx et al., 2022) and in mice after SE (Chauvière et al., 2009; Hort et al., 1999; Mikati et al., 2001; Müller et al., 2009). Two different cognitive tests (Barnes Maze, Morris Water Maze; see below), both of which probe the same underlying processes, and substrates were used to assess spatial memory function to avoid carryover effects of repeated testing (Dellu et al., 1997; Gawel et al., 2019; Heimer‐McGinn et al., 2020; Markowska & Savonenko, 2002; Pitsikas et al., 1991). AnyMaze software was used for video tracking.

### 2.6 Barnes Maze

The Barnes Maze (BM) is used to study spatial memory and navigation, and testing was conducted as we have previously described (Caccavano et al., 2020). The maze consists of an elevated circular platform (92 cm) with 20 holes (5 cm) around the perimeter (Maze Engineers). The target hole consisted of an escape box, and the remaining holes had a false floor. The location of the escape box remained consistent, and animals used spatial cues to find the escape platform. Aversive overhead lighting and radio static were used to motivate the mice to find the escape platform. Animals were trained for four consecutive days, four trials each day with an intertrial interval of 15 min. Each trial was 180 s long. If the animal failed to find the target hole, it was guided to the escape hole. Animals were kept in the escape box for 30 s. Twenty‐four hours following the fourth training day, animals were subjected to the probe trial, where they were given 90 s to find the escape platform. The chamber was cleaned thoroughly between animals. Latency to the platform was used to measure learning each day during training and for probes.

### 2.7 Morris Water Maze

The Morris Water Maze (MWM) is used to study spatial memory and navigation. Testing was done as we have previously described (Speidell et al., 2019; Washington et al., 2012; Wurzman et al., 2014). The maze consists of a large pool (4 feet) filled with water, made opaque with nontoxic Crayola paint to hide the escape platform. The escape platform remains hidden in a fixed position. Animals are placed in the water at various starting points and must use spatial cues around the maze to find the escape platform. Animals were trained for 4 days, 2 trials 30 min apart each day. Animals were taken to the escape platform if they were unable to find the platform after 90 s. After each trial, the animals were placed on a heating pad to dry off. Twenty‐four hours after the fourth training day, animals were tested for the probe, and they were given 90 s to find the escape platform. The latency to reach the platform was measured.

### 2.8 Immunohistochemistry

Mice were anesthetized and perfused transcardially with 1× PBS followed by 4% paraformaldehyde (PFA) in PBS. Brains were removed and postfixed in 4% PFA overnight, then cryoprotected in 30% sucrose in 0.1 M PBS. Brains were flash‐frozen and sectioned at 30 μm thickness on a cryostat (Leica CM 1850).

For staining, slides were washed with 1× PBS with 0.1% Triton‐x 100 3 times for 5 min each. Nonspecific binding was blocked with Animal‐Free Blocker and Diluent (Vector Laboratories, SP‐5035‐100) for 1 h at room temperature. Primary antibodies in this study were mouse‐anti‐p16 (Abcam ab54210), mouse‐anti‐p21 (Santa Cruz, sc‐6246) both used at 1:500 in blocking solution. Ab54210 has previously been validated and does not produce staining in the liver from p16 knockout animals (Safwan‐Zaiter et al., 2022). Sc‐6246 has also previously been validated and does not produce labeling on western blot in cell lines lacking p21 (Galanos et al., 2018). Antibodies were incubated overnight at 4°C. On the second day, the primary was washed off three times in PBS. The slides were incubated with Alexa Fluor conjugated secondaries at 1:1000 (Jackson ImmunoResearch Laboratories Inc) for 1 h at room temperature. The tissues were washed three times for 5 min each with PBS, quenched for autofluorescence (Vector Laboratories, SP8400‐15), and cover‐slipped with VECTASHIELD Vibrance® Antifade Mounting Medium with DAPI (Vector Laboratories, H‐1800). Sections were observed by fluorescence microscopy.

### 2.9 SPiDER‐β‐gal

For the SPiDER‐β‐gal stain, tissue sections were incubated in 20 μM SPiDER‐β‐gal (Dojindo, SG02‐10) in solution in 0.1% Triton in PBS for 60 min at 37°C. After washing of tissue sections, nuclei were stained with DAPI, and tissue sections were mounted with VECTASHIELD Vibrance® Antifade Mounting Medium with DAPI (Vector Laboratories, H‐1800). Sections were observed by fluorescence microscopy.

### 2.10 Microscopic analysis and quantification

Widefield images of the hippocampus were acquired on the Leica Mica confocal workstation at 20× magnification. Three maximum projections of 18 μm z‐stack images of hippocampi were analyzed per animal. QuPath software was used to quantify immunoreactive cells per hippocampal area using the single measurement threshold object classifier (Bankhead et al., 2017).

### 2.11 Blinding

All data and analyses were collected and performed by observers who were blinded to the treatment status and age of the animals.

### 2.12 Statistics

All data are expressed as mean ± SEM. Experimental groups were assigned by simple randomization. Graphpad prism (version 10.0.2) software was used for statistical analysis. Seizure data were analyzed using Kruskal–Wallis test, followed by Dunn's multiple comparison test. Survival curves were analyzed by log‐rank (Mantel‐Cox) tests. Behavioral training trials were analyzed by two‐way ANOVA, followed by Holms‐Šidák's multiple comparison test, and probe trials were analyzed by two‐tailed unpaired *t*‐test for data with equal variances. Histological analysis was analyzed by one‐way ANOVA.

## 3 RESULTS

### 3.1 Seizure susceptibility reduced with D&Q pretreatment

**FIGURE 1:**
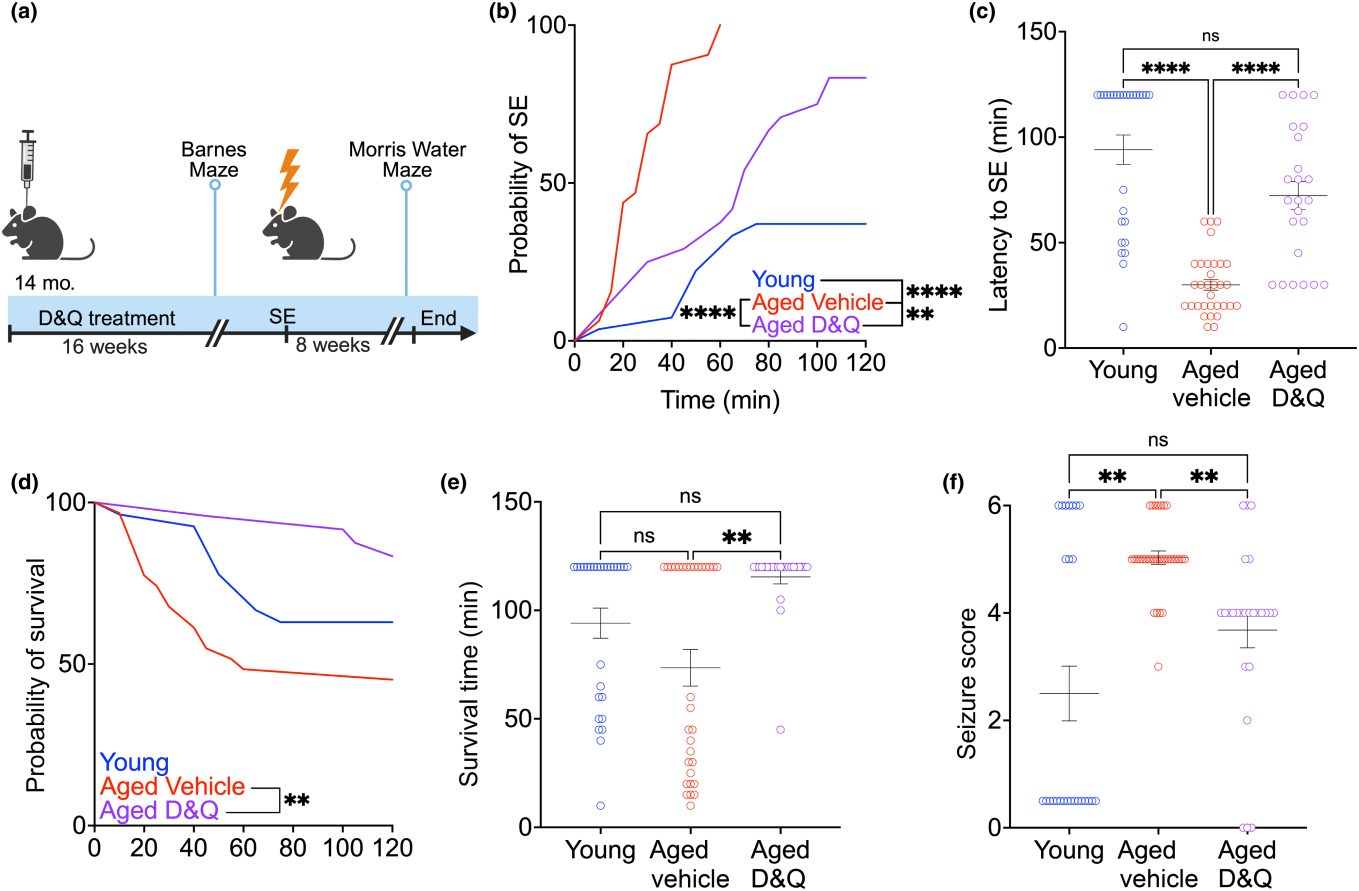
Effects of prophylactic senolytic treatment on seizure burden. (a) Schematic of experimental procedure. Fourteen‐month‐old mice were randomly assigned to once‐weekly D&Q (*n* = 24) or VEH treatment (*n* = 32). After 16 weeks of D&Q or VEH treatment, aged mice were subjected to Barnes Maze to assess spatial memory. Following Barnes Maze, aged and young (*n* = 28) animals underwent *Status Epilepticus* (*SE*) for 120 min by PILO (260 mg/kg). Eight weeks following *SE*, aged mice were subjected to Morris Water Maze to assess the effects of senolytic treatment on spatial memory following epilepsy development. Created with Biorender.com (ZW26RQFQIT). (b) Probability of animals entering SE for 120 min of young, and aged VEH‐treated, and aged D&Q treated. Aged D&Q‐treated mice had a reduced latency to *SE* compared to young. (c) Latency to *SE*. (d) Probability of survival during *SE*. (e) Survival time during *SE*. (f) Maximal seizure score of animals during *SE*; significant difference between groups. For all: **p < 0.01, ****p < 0.0001. (b, d) Log‐rank (Mantel‐Cox) test; (c, d, f) Kruskal–Wallis, Dunn's multiple comparison test. Individual points represent individual animals. Line and error bars indicate mean ± SEM.

We treated *n* = 24 14‐month‐old C57BL/6J mice with an established senolytic therapy, consisting of a combination of Dasatinib (5 mg/kg) and Quercetin (50 mg/kg) (D&Q), administered once per week (Ogrodnik et al., 2019; Zhang et al., 2019; Zhu et al., 2015). Age‐matched animals whose senescent cells remained received vehicle treatment (*n* = 32). After 16 weeks of D&Q, aged mice were injected with PILO to induce *SE* (Turski et al., 1984) (Figure 1a).

We used a lower dose of PILO, 260 mg/kg, to reduce mortality, as aged animals have a higher susceptibility to seizures (Cavalheiro et al., 1987; Hirvonen et al., 1993; McCord et al., 2008). This dose has not been previously successful in our lab to induce SE in young mice (<6 month age) of the same background. Consistent with this, in another chemoconvulsant model of epilepsy, the kainic acid (KA) model of acute seizures, aged C57BL/6J mice received half of the KA dose as young mice and had higher reactive gliosis and neurodegeneration (Benkovic et al., 2006). Animals were monitored for 2 h after PILO administration, after which seizures were terminated with diazepam (5 mg/kg). Young animals were included in this study for comparison (*n* = 28). Onset of SE was defined as 5 min of uninterrupted bilateral forelimb clonus (Stage 3 seizures).

As expected, aged VEH‐treated mice had a significantly reduced latency to SE compared to young and aged D&Q‐treated mice, and aged D&Q‐treated mice had a significantly reduced latency to SE compared to young. 100% of VEH aged mice reached SE, compared to 83% of D&Q aged mice and 37% of young mice that reached SE (Young and age‐matched D&Q‐treated mice: *χ* ^2^ = 43.54, *p* < 0.0001; *χ* ^2^ = 31.1, *p* < 0.0001, respectively; Mantel‐Cox test. Aged D&Q‐treated mice had a reduced latency to SE compared to young: *χ* ^2^ = 8.21, *p* = 0.0042; Mantel‐Cox Test.) (Figure 1b). The latency to SE was significantly lower in VEH‐treated aged mice (30 min after PILO administration), while a later onset was observed in young mice (94 min, *p* < 0.0001), and D&Q‐treated aged mice (72 min, *p* < 0.0001) (*H* = 42.76, *p* < 0.0001, Kruskal–Wallis test, Dunn's test, Figure 1c).

We further examined the survival of young and aged mice during SE (*H* = 12.5, *p* = 0.0019, Kruskal–Wallis test). Interestingly, the young mice that survived did not reach SE. Survival duration differed between groups, driven by shorter survival in aged, VEH‐treated mice (74 min) as compared to aged, D&Q‐treated mice (115 min, *p* = 0.0013, Dunn's test), with no difference between aged D&Q mice and young mice (36 min; *p* = 0.26, Dunn's test; Figure 1d,e). Senolytic therapy also reduced behavioral seizure severity; the median behavioral seizure score was highest in VEH‐treated aged mice (5) compared to aged D&Q‐treated (4, *p* = 0.003), and young (0.5, *p* = 0.0016) (*H* = 15.79, *p* = 0.0004, Kruskal‐Wallis test, Figure 1f). Our findings indicate that D&Q treatment caused a shift in survival and resilience during SE, suggesting a protective effect of senolytic therapy.

### 3.2 Senolytic therapy normalizes age‐associated cognitive dysfunction prior to SE

Clearance of senescent cells improves cognitive impairments during aging (Ogrodnik et al., 2021). Further, D&Q treatment improved cognitive function in rats and increased dendritic spine length and size on apical dendrites of CA1 neurons (Krzystyniak et al., 2022). As cognitive functions are compromised in aging and epilepsy, we evaluated the effects of prophylactic senolytic treatment on cognitive function both before and after SE (after epilepsy development).

**FIGURE 2:**
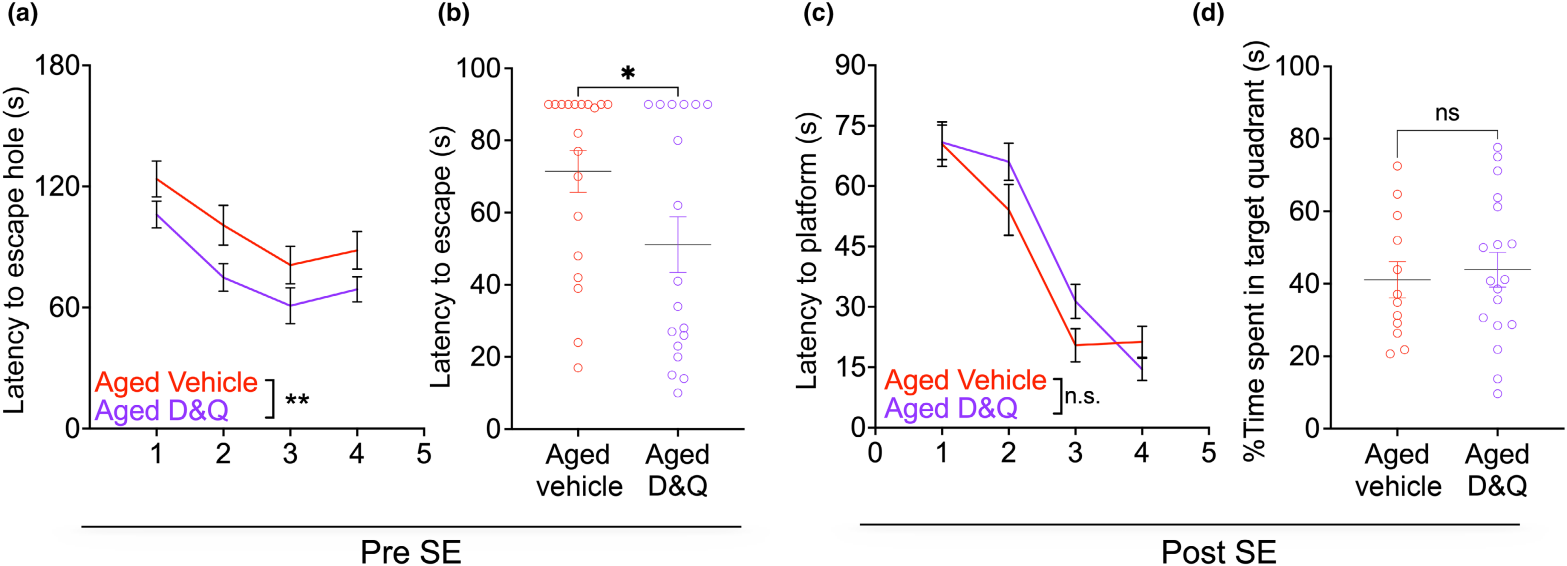
Effects of prophylactic senolytic treatment on spatial memory. (a) Barnes maze training consisted of 4 days with 4 × 180 s trials each day, with an intertrial interval of 15 min. (b) During the probe trial 24 h after the last training session, animals were on the platform for one 90‐s trial in which latency to find the escape platform was measured. (c) Morris Water Maze training consisted of 4 days with two 90‐s trials each day, with an intertrial interval of 15 min. (d) There was no difference between the groups on the probe trial. For all: *p < 0.05, **p < 0.01. Training (a, c) two‐way ANOVA, repeated measures analysis, Holms‐Šidák's multiple comparison test, and probe (b, d) Unpaired two‐tailed *t*‐test. Individual points represent individual animals. Line and error bars indicate mean ± SEM.

After 16 weeks of D&Q or VEH treatment and prior to SE, we assessed spatial memory with the Barnes Maze (BM) test in which mice use spatial cues to find an escape box. D&Q treated, aged mice learned faster in the BM as evident by the significant main effect of treatment and training day (*F*_(1,35)_ = 10.79, *p* = 0.0023 and *F*_(3,105)_ = 10.83, *p* < 0.0001, respectively, two‐way RM ANOVA) over four training (learning) days. There was no treatment‐by‐training day interaction (*F*_(3,105)_ = 0.095, *p* = 0.96; Figure 2a). Twenty‐four hours after the fourth training day, aged VEH‐treated and aged D&Q‐treated mice were tested for the probe trial to test memory retention. VEH‐treated aged mice required a significantly longer time to find the escape platform (*t* = 2.12, df = 35, *p* = 0.041, Unpaired two‐tailed *t*‐test, Figure 2b). Latency to the escape platform was significantly lower in aged D&Q‐treated (51 s) compared to aged VEH‐treated mice (71 s).

### 3.3 Senolytic therapy does not prevent SE‐associated cognitive impairment in aged mice

We were interested in whether prophylactic senolytic treatment preserves spatial memory in aged mice after epilepsy development. In the PILO model, mice typically develop spontaneous recurrent seizures 2–4 weeks after SE. In addition to age‐associated cognitive deficits, recurrent seizures can also worsen cognitive function with hippocampal sclerosis, neuronal loss, inflammation, and aberrant hippocampal circuitry rewiring (Hwang et al., 2020). We continued D&Q or VEH treatment for 8 weeks after SE (during which time we typically see spontaneous recurrent seizure development) and assessed spatial memory using the MWM of the surviving animals. We used two different spatial memory tests to avoid carryover effects.

Similar to BM, animals were trained for 4 days on the MWM followed by a probe test conducted 24 h after the last training session. There was a main effect of training day (*F*_(2.818,78.89)_ = 68.89, *p* < 0.0001, two‐way RM ANOVA, Figure 2c), but neither a main effect of treatment (*F*_(1,28)_ = 1.305, *p* = 0.26) nor a treatment‐by‐training day interaction (*F*_(3,84)_ = 2.11, *p* = 0.105). There was also no difference between the groups on the probe trial (*t* = 0.389, df = 28, *p* = 0.7, unpaired two‐tailed t‐test, Figure 2d).

### 3.4 Senescent cell expression following senolytic treatment

**FIGURE 3:**
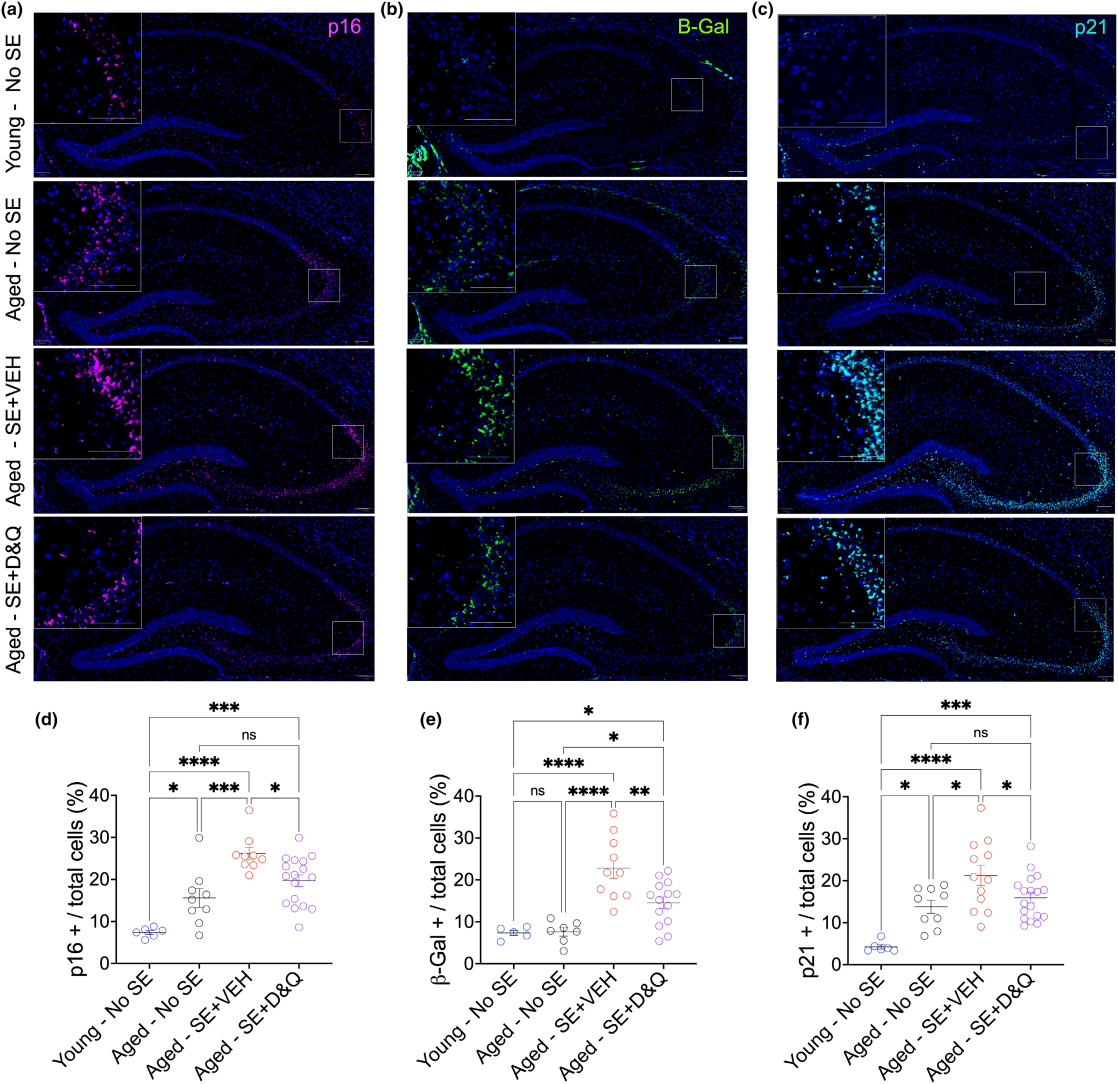
Senescent cell expression profile in hippocampus. (a) Representative images of p16 expression, (b) senescence‐associated β‐galactosidase expression, and (c) p21 expression from hippocampus in young No *Status Epilepticus*, aged No *SE*, aged‐VEH‐treated mice 8 weeks following *SE*, and aged D&Q‐treated mice following *SE*. (d) Percent of p16‐positive cells, (e) senescence‐associated beta‐galactosidase positive cells, and (f) p21‐positive cells over total detected cells in the hippocampus. For all: *p < 0.05, **p < 0.01, ***p < 0.001, ****p < 0.0001, ordinary one‐way ANOVA, Holms‐Šidák's multiple comparison test. Individual points represent individual animals. Line and error bars indicate mean ± SEM.

Following completion of behavioral experiments, we examined the expression of proteins that are upregulated in senescent cells: p16^INK4a^, p21^CIP1/WAF1^, and senescence‐associated β‐galactosidase (β‐gal) activity. We focused our analyses on the hippocampus as recurrent seizures and aging impact memory processes (Figure 3). All histological data were analyzed by one‐way ANOVA followed by Holms‐Šidák's multiple comparison test (p16: *F*_(3,37)_ = 11, β‐gal: *F*_(3,32)_ = 14.80, p21: *F*_(3,41)_ = 11.47).

As expected, p16 expression increased with age (young vs. aged No SE: *p* = 0.0263) and further increased following SE in aged mice (aged SE + VEH vs. young: *p* < 0.0001 vs. age‐matched controls: *p* = 0.0008). Senolytic treatment reduced p16 expression following SE to normal aging levels (aged SE + D&Q vs. aged No SE: *p* = 0.07 vs. aged SE + VEH: *p* = 0.0185), but not to that of young mice (*p* = 0.0005) (Figure 3d).

β‐gal expression also did not differ with aging but was elevated during epilepsy development in aged mice (both aged SE + VEH vs. young and vs. aged control: *p* < 0.0001). Senolytic treatment reduced β‐gal expression following SE (*p* = 0.003), though it remained higher than normal aging levels (*p* = 0.026) and young (0.03) (Figure 3e).

p21 expression increased with age (*p* = 0.014). SE in aged mice further exacerbated p21 expression compared to young (aged SE + VEH vs. young: *p* < 0.0001) and age‐matched controls (aged SE + VEH vs. aged: *p* = 0.01). Senolytic treatment reduced p21 expression following SE (vs. aged SE + VEH: *p* = 0.039) to normal aging levels but not to the level of young mice (*p* = 0.0007) (Figure 3f).

### 3.5 Sex differences

We did not detect any appreciable sex differences in this study, so we collapsed across sex for our primary analyses. Analyses including sex as a factor are presented in Appendix S1.

## 4 DISCUSSION

This is the first study to target senescent cells as a preventative strategy for seizures in aged populations. The main findings in this present study are as follows: (a) senolytic therapy with D&Q reduced mortality during SE and seizure severity; (b) prophylactic senolytic therapy improves spatial memory deficits before seizures but does not rescue spatial memory deficits after seizure insults; and (c) seizures increase senescent cell expression in aged animals, while continuous senescent cell ablation reduces senescence pathology in the hippocampus. These findings indicate that senescent cell accumulation can increase the risk of seizures, and targeting these cells may reduce or prevent seizure incidents that increase with aging.

### 4.1 Seizure susceptibility reduced with D&Q pretreatment

It is well recognized that senescent cells accumulate in aging populations and that populations over 60 years of age have a high incidence of seizures and epilepsy (del Pozo et al., 2022). However, no prior studies have linked senescence to seizures in the aged population. Quercetin alone has been shown to be neuroprotective against seizures, however, this has only been studied in young mice (Prakash et al., 2023). Our findings are the first to demonstrate that senolytics have the potential to reduce seizure incidence in aged populations.

In this study, we evaluated seizure susceptibility during SE in aged mice pretreated with senolytics, D&Q. Despite using a lower dose of PILO in our study, the VEH‐treated aged mice had the highest mortality rate of all groups; however, there were no significant differences in mortality between young and aged D&Q‐treated mice. Our findings indicate that D&Q treatment caused a shift in survival and resilience during SE, suggesting a protective effect of D&Q against hyperexcitability in the aged brain. A limitation of this study was that spontaneous recurrent seizures were not evaluated after SE to determine whether senescent cell ablation can prevent epilepsy development. However, this is a promising avenue for seizure prevention and therapy in the aged population.

### 4.2 Cognitive dysfunction restored after senolytic treatment, but not restored after SE

The relationship between senescent cell accumulation and heightened seizure vulnerability in the aging brain has not been studied before. Our findings demonstrated that D&Q treatment in aged mice did not rescue spatial memory deficits following epilepsy development on MWM. Previous studies have demonstrated impaired performance by aged epileptic rats on MWM (Cavarsan et al., 2013). These results are not surprising as SE causes hippocampal degeneration (Pitkänen et al., 2002). It is possible that SE caused irreversible damage in aged mice that post‐SE therapeutic interventions could not attenuate. Further, we based our dosing on published aging literature, and a higher dose of senolytics may be necessary to attenuate senescent cell burden in aged animals after an insult such as SE. Other studies have used higher doses of quercetin (100 mg/kg) for epilepsy treatment in young animals (Moghbelinejad et al., 2016). Though senolytic treatment may not have rescued hippocampal‐dependent spatial memory after epilepsy, cognitive processes such as anxiety remain to be explored. Senescent cell clearance has alleviated anxiety‐like behavior in a model of obesity (Ogrodnik et al., 2019).

### 4.3 Senescent cell expression following senolytic treatment

Global senolytic treatment affects all tissues as they broadly target apoptotic pathways. While our study focused specifically on the brain, others have shown that senescent cell clearance reduced age‐related degeneration in various tissues such as kidneys, cardiac tissue, and sarcopenia (Baker et al., 2011, 2016). Genetic tools to clear senescent cells in specific cells/tissues only recently became readily available and demonstrated that local senolysis in aged tissue partially replicates the benefits of systemic senolysis (Farr et al., 2023). Several studies have demonstrated that senescent cells increase in the brain in normal aging (Baker & Petersen, 2018; Zhu et al., 2015). Microglial senescence has been identified in 26–29‐month‐old mice using a flow cytometry approach (Talma et al., 2021), and in 24–29‐month‐old animals using a single‐cell RNA‐sequencing and histopathological approach (Ogrodnik et al., 2021; Zhang et al., 2022). Similarly, increased p16 expression was reported in human microglia from aged healthy donors (Gerrits et al., 2021; Talma et al., 2021). Spatial transcriptomic profiling likewise has demonstrated increased myeloid lineage senescence in the cortex and hippocampus of 22–24‐month‐old mice (Carver & Schafer, 2022). Senescent‐like microglia in aged animals demonstrate elevated transcript levels for a range of SASPs (Stojiljkovic et al., 2022), including IL‐10, IL1B, IL‐6, TNFA, and BDNF, all of which may contribute to epilepsy and seizure vulnerability (Vezzani et al., 2017; Vezzani & Granata, 2005). Moreover, microglia in aging and neurodegeneration display a disease‐associated microglia signature which overlaps with the SASP and includes upregulated *Ccl3, Ccl4, Ccl6, Cd52, Apoe*, and *Fth1* in addition to *Cdkn1a* which in turn promotes increased expression of senescence markers such as p21 (Choi et al., 2023). In addition to microglial senescence, senescent astrocytes have been reported in aged human hippocampi (Matias et al., 2022). Moreover, senescent neural precursor cells were found in the hippocampi of 12‐month‐old mice and contributed to declined neurogenesis (Fatt et al., 2022; Micheli et al., 2019; Molofsky et al., 2006). Collectively, these studies point toward a diverse and multifaceted role of senescence in the aging brain, manifesting distinct cell types that are primarily glial cells. While the mechanisms by which clearing senescent cells with D&Q reduced seizure susceptibility in aged animals remain unknown, senolytic therapy—which reduces SASP production—would likely reduce age‐associated oxidative stress and inflammation that exacerbates hyperexcitability during seizures.

## 5 CONCLUSIONS

In summary, removing senescent cells improves hippocampal‐dependent memory and protects against enhanced vulnerability to *SE* in aged mice. To our knowledge, we are the first to utilize senolytic therapy to prevent seizure susceptibility in aged mice. There are over 20 completed, ongoing, or planned clinical trials of senolytic therapies. The first senolytic clinical trial in an open‐label study of IPF was treated with intermittent D&Q, which suggested improved physical function and no serious adverse effects (Nambiar et al., 2023). As certain anti‐seizure medications (ASMs) interfere with memory processes as well as other side effects, senolytics pose a promising treatment to prevent seizures and associated cognitive dysfunction in aged populations.

## AUTHOR CONTRIBUTIONS

Conceptualization: T.K. and P.A.F.; Data curation: P.A.F.; Formal analysis: T.K., A.I.H., T.P.C., L.F., M.C., and P.A.F.; Funding acquisition: T.K. and P.A.F.; Investigation: T.K., A.I.H., T.P.C., L.F., M.C., G.W., and D.M.; Project administration: T.K. and P.A.F.; Resources: P.A.F.; Supervision: T.K. and P.A.F.; Visualization: T.K. and P.A.F.; Writing—original draft: T.K.; Writing—review and editing: T.K., A.I.H., T.P.C., L.F., M.C., G.W., D.M., and P.A.F.

## FUNDING INFORMATION

This work was supported by R21NS125552 to PAF. TK was supported by F99NS129108 and T32NS041218. DM was supported by T32GM142520. AIH was supported by T32GM144880.

## CONFLICT OF INTEREST STATEMENT

The authors have no conflicts to disclose.

## Supporting information


Appendix S1.

## Data Availability

Data are available upon request from the authors.
