# Supplementary material for: Prophylactic senolytic treatment in aged mice reduces seizure severity and improves survival from Status Epilepticus
Source: Aging Cell. 2024 Jun 20;23(9):e14239. doi: 10.1111/acel.14239 (PMC11488304; doi:10.1111/acel.14239)
Supplement: Supplementary file 1 — Appendix S1. [file ACEL-23-e14239-s001.docx]

**Supplementary Results**

*Sex differences.* All analyses were performed on sex balanced populations. Young males had a higher probability of reaching SE than females (X^2^ = 7.45, p=0.0064; Mantel-Cox Test), whereas aged vehicle-treated females had a higher probability than males for reaching SE (X^2^ = 4.28, p=0.04; Mantel-Cox Test). There were no sex differences in SE probability for aged-D&Q treated mice. Sex differences were not a factor in the latency to SE (H=45.85, Kruskal-Wallis Test, Dunn’s multiple comparison test). Young females displayed a higher probability of survival (X^2^ = 18.54, p=0.0023; Mantel-Cox Test) otherwise no other group displayed apparent sex differences in the probability of survival. There were no differences between groups for survival time. Young males had a higher maximal seizure score during SE than females (H=20.54, p=0.0166 Kruskal-Wallis Test, Dunn’s multiple comparison test). There were no sex differences in maximal seizure score for aged vehicle-treated or D&Q-treated mice. There was a main effect of day, treatment, and sex and day interaction for Barnes Maze training (F_(3,99)=8.8_, p<0.0001, F_(1,33)=7.9_, and p=0.0081, F_(3,99)=3.1_, and p=0.0271, respectively; Three-way ANOVA), however, there were no sex differences for Barnes Maze training. There was only a main effect of trial day for Morris Water Maze training (F_(3,78)=40_; p<0.0001, Three-way ANOVA), but there were no effect of sex, treatment, or any interaction. There were no sex differences in the probe trial for either test (H=3.519, p=0.318 and H=0.435, p=0.933; Kruskal-Wallis Test, respectively). There were no main effect of sex differences in percent of p16 (F_(1,33)=0.4_, p=0.499), senescence-associated beta-galactosidase (F_(1,27)=.1_, p=0.687), and p21 positive cells (F_(1,37)=1.1_, p=0.284) over total detected cells in the hippocampus (Two-way ANOVA).
